# The roles and molecular mechanisms of non-coding RNA in cancer metabolic reprogramming

**DOI:** 10.1186/s12935-023-03186-0

**Published:** 2024-01-18

**Authors:** Shizhen Li, Mingjing Peng, Shiming Tan, Linda Oyang, Jinguan Lin, Longzheng Xia, Jiewen Wang, Nayiyuan Wu, Xianjie Jiang, Qiu Peng, Yujuan Zhou, Qianjin Liao

**Affiliations:** 1 Hunan Key Laboratory of Cancer Metabolism, Hunan Cancer Hospital and the Affiliated Cancer Hospital of Xiangya School of Medicine, Central South University, 283 Tongzipo Road, Changsha, 410013 Hunan China; 2 Hengyang Medical School, University of South China, Hengyang, 421001 Hunan China

**Keywords:** NcRNAs, Metabolic reprogramming, Cancer therapeutic tolerance

## Abstract

One of the key features of cancer is energy metabolic reprogramming which is tightly related to cancer proliferation, invasion, metastasis, and chemotherapy resistance. NcRNAs are a class of RNAs having no protein-coding potential and mainly include microRNAs, lncRNAs and circRNAs. Accumulated evidence has suggested that ncRNAs play an essential role in regulating cancer metabolic reprogramming, and the altered metabolic networks mediated by ncRNAs primarily drive carcinogenesis by regulating the expression of metabolic enzymes and transporter proteins. Importantly, accumulated research has revealed that dysregulated ncRNAs mediate metabolic reprogramming contributing to the generation of therapeutic tolerance. Elucidating the molecular mechanism of ncRNAs in cancer metabolic reprogramming can provide promising metabolism-related therapeutic targets for treatment as well as overcome therapeutic tolerance. In conclusion, this review updates the latest molecular mechanisms of ncRNAs related to cancer metabolic reprogramming.

## Introduction

Cancer is one of the most common fatal diseases, which seriously threatens human lives and health. According to the most recent data in 2023, about two million new cancer cases and almost sixty thousand cancer deaths are projected to occur in the United States [1]. Thus, it is urgent to find new biomarkers and therapeutic strategies for cancer with great significance.

Currently, energy metabolic reprogramming, one of the Hallmarks of cancer, has become the hot spot in the research field [2]. In 1921, German scientists discovered the “Warburg effect”, also known as “aerobic glycolysis”, which meant that apart from normal cellular oxidative phosphorylation, cancer cells undergo aerobic glycolysis under aerobic conditions by taking in massive glucose to provide numerous Adenosine triphosphate (ATP) and biosynthetic materials for rapid proliferation. It is a huge breakthrough in the field of cancer metabolism research. Besides glucose metabolism reprogramming, cancer cells can also induce the alterations of lipids and amino acids et al*.* to meet the requirements for malignant progress. The altered energy metabolism of cancer cells can reshape the cancer microenvironment, which facilitates the maintenance of malignant phenotype and immune escape [3, 4]. Importantly, targeting specific metabolic pathways of cancer cells or simultaneously synergizing other therapeutic strategies is one of the effective means to improve the efficacy of cancer therapy [5, 6].

Non-coding RNAs (ncRNAs) are novel gene regulatory molecules, mainly including miRNAs, long non-coding RNAs (lncRNAs) and circular non-coding RNA (circRNAs) et al. In recent years available evidence is increasingly indicating that dysregulated ncRNAs play a significant role in the progression of cancer malignancy through metabolic reprogramming [7, 8]. Consequently, there is a pressing need to elucidate the potential regulatory mechanisms of ncRNAs in the cancer metabolic network for the development of carcinogenesis, as this knowledge holds great promise for future prevention and treatment strategies. This review aims to provide an updated overview of the latest research progress on the molecular mechanisms of ncRNAs in cancer energy metabolic reprogramming.

## Overview of metabolic reprogramming in cancer cells

It is known that energy metabolism undergoes great changes during the development of cancer, referring to the “Warburg effect”, the production of vast amounts of ATP and lactate by aerobic glycolysis of cancer cells under aerobic conditions through massive glucose uptake to support the acquisition and maintenance the malignant phenotype [9]. The initiation of aerobic glycolysis is facilitated by the glucose transporter1 (GLUT1) mediated abnormal transportation of glucose and the abnormal expression of enzymes involved in glycolysis. This phenomenon is induced by oncogenes like c-Myc and HIF-1α, whereas cancer suppressors such as p53 and OVOL2 exhibit an opposing effect. The activation of aerobic glycolysis results in the provision of substantial resources for cancer cells, encompassing ATP and metabolic intermediates derived [10–15]. In addition to facilitating the malignant phenotype of cancer cells, lactate, a byproduct of aerobic glycolysis in cancer cells, can be extracellularly transported through monocarboxylate transporter protein 4. This transport mechanism contributes to the creation of an acidic microenvironment within cancer cells, ultimately leading to the polarization of M2 macrophages and the promotion of immunosuppressive function in Treg cells [3]; Conversely, it inhibits the activation of cytotoxic T cells and depletes the interferon-γ (IFN-γ) secretion function of natural killer (NK) cells [3, 16, 17]. Furthermore, increased lactate abundance in the tumor microenvironment (TME) also promotes cancer immune escape through epigenetics [18]. In conclusion, glucose metabolic reprogramming promotes the malignant progression of cancer cells and its targeting can be a promising strategy to suppress cancer progression.

The mechanisms of lipids including absorption, synthesis, and oxidation in cancer cells primarily distinct from normal cells [19]. These processes are extensively regulated by transcription factors such as peroxisome proliferator-activated receptors and sterol regulatory progenitor proteins [20, 21]. Cancer cells require a large amount of lipids and cholesterol to meet their raised demand by increasing the uptake of exogenous lipids, and lipoproteins and activating the expression of enzymes related to the de novo synthesis of lipids and cholesterol. The increased expression of fatty acids in vivo can modify proteins at the epistatic level. An emerging study shows that palmitoylation modification of GLUT1 regulates its plasma membrane localization impairing glycolysis and cell proliferation in cancer cells [22]. Furthermore, ferroptosis is driven by the peroxidation of polyunsaturated fatty acids catalyzed by ferrous ions [23]. Changes in lipid metabolism in cancer cells promote aberrant accumulation of lipids in TME, which in turn promotes unusual accumulation of lipids in cancer-infiltrating immune cells leading to the translation between immunosuppressive and anti-inflammatory phenotype [5, 24–26]. Intriguingly, the abnormal accumulation of cholesterol in cancer cells can contribute to the stable expression of programmed death ligand-1 (PD-L1) by binding to cholesterol-recognition amino acid consensus motifs on PD-L1, resulting in the development of cancer immune escape [5]. As its low response to immunotherapy, targeting lipid metabolism in cancer may be a significant approach to improve the efficiency of immune therapy and inhibit cancer progression.

The metabolic reprogramming of amino acids plays an essential role in the development of cancer. In addition, amino acids affect the function of immune cells in anti-cancer immunity [27]. Accumulated evidence shows that most cancer cells strongly depend on glutamine known as “glutamine addiction” as it stagnates in growth and even dies once cancer cells are deprived of glutamine [28]. Cancer cells reduce the activation and infiltration of CD8^+^ T cells and recruit myeloid-derived suppressor cells (MDSCs) by increasing the secretion of granulocyte colony-stimulating factor through the massive consumption of glutamine in TME [3]. However, restricting glutamine uptake by cancer cells increases PD-L1 expression through stimulation of Ca2^+^/NF-κB signaling, resulting in diminished anticancer activity of T cells, which may be successfully eliminated by anti-PD-L1 therapy [6]. The altered metabolism of serine, tryptophan and glycine in cancer cells has likewise received considerable attention. The classically activated macrophages (M1) polarization of macrophages induced by IFN-γ can be significantly enhanced by inhibiting the ab initio synthesis of serine or by limiting the uptake of exogenous serine [29]. Serine is the main substrate of one-carbon units for nucleotide synthesis in vivo, however, in cancer cells with high expression of indoleamine 2, 3-dioxygenase 1(IDO1), tryptophan could replace serine as the main substrate of one-carbon units [30]. High levels of IDO and tryptophan 2,3-dioxygenase (TDO) in cancer inhibit the cancer-killing effects of T cells by reducing tryptophan levels in TME [31]. Related studies have proved that immunotherapy induces IFN-γ increasing release by activation of CD8^+^ T cells, downregulates the expression of the xc-subunit of the glutamate-cystine reverse transport system and weakens the uptake of cystine by cancer cells, thereby promoting cancer cell death [32]. Therefore, it may have the potential to inhibit the malignant progression of cancer cells as well as improve the efficacy of anticancer therapy by targeting specific amino acid metabolic pathways.

In addition, aberrant activation of oncogenes can promote anabolism through multiple signaling pathways, such as PI3K/Akt/mTOR, HIF-1α and sterol-regulatory element binding proteins (SREBP) et al. [33]. TME provides the fundamental materials for cancer growth, and cancer metabolic reprogramming changes the level of substances in TME, indirectly affecting the release of inflammatory factors and anti-cancer function of immune cells in TME [34]. In conclusion, as metabolic reprogramming of cancer cells facilitates the development of metabolic phenotype for malignant progression. it may be a potential strategy to effectively suppress their malignant progression by changing the metabolic phenotype of cancer.

## Overview of NcRNAs

For decades, ncRNAs, used to be regarded as by-products of transcription, have been found to play essential roles in a variety of important cellular life activities, which include chromatin remodeling, transcription, post-transcriptional modifications (PTMs) and signal transduction [35–39]. NcRNAs refer to a class of RNAs that have no protein-coding functions in cells, including ribosomal RNA (rRNA), transfer RNA (tRNA), small nuclear RNA (snRNA), and small nucleolar RNA (snoRNA), piRNA (PIWI-interacting RNA), microRNA (miRNA), lncRNAs, and circ RNAs [40, 41]. In this review, we mainly focus on microRNAs, lncRNAs and circ RNAs.

MicroRNAs are a set of ncRNAs with 20nt nucleotide and play a key regulatory role at the post-transcriptional level of messenger RNA (mRNA) by binding to the 3’-untranslated regions (3’-UTR) of the target gene to degrade the mRNA of the target gene or inhibit the translation process of the target gene [42, 43]. LncRNAs are a class of special RNA molecules that are longer than 200 nucleotides and without protein-coding potential, and their biological generation is similar to mRNAs [44]. However, due to the splicing efficiency of lncRNAs being lower than mRNAs, most lncRNAs are retained in the nucleus after transcription involved in mediating interactions between DNA and proteins, adsorbing microRNAs, and binding to proteins as decoys, which contribute to regulating chromatin structure, remodeling chromatin and chromatin modification. And only a small fraction is exported to the cytoplasm [45–47]. CircRNAs are novel ring-like molecules formed by reverse shearing of exons. The 3’ and 5’ ends of circ RNA are linked to form a covalently closed loop structure, which confers a longer half-life, more stability and less sensitivity to RNA exonucleases. Similar to lncRNAs, circRNAs have played an important regulatory role in gene expression at epigenetic, transcriptional or post-transcriptional levels [48–50]. There is sufficient evidence showing that ncRNAs are key regulators of metabolic reprogramming, which results in cancer rapid development [51–53]. Importantly, some ncRNAs serve as metabolic-related oncogenes, while some other ncRNAs act as metabolic-related suppressors. Thus, it is urgent to figure out the potential molecule mechanism of dysregulated metabolism mediated by ncRNAs in carcinogenesis, which may be a promising strategy for therapy in the future.

## NcRNAs affect cancer progression through metabolic reprogramming

Numerous research has demonstrated that dysregulated ncRNAs related to metabolic networks can promote or suppress carcinogenesis, by facilitating cancer to acquire and maintain aggressive malignant phenotype and to target multiple metabolic signaling pathways [54–62]. Therefore, we update the latest research progress on the mechanisms of ncRNAs in metabolic reprogramming from three perspectives: glucose, lipid and amino acids, respectively.

### The effects of ncRNAs on cancer glucose metabolic reprogramming

**Fig. 1:**
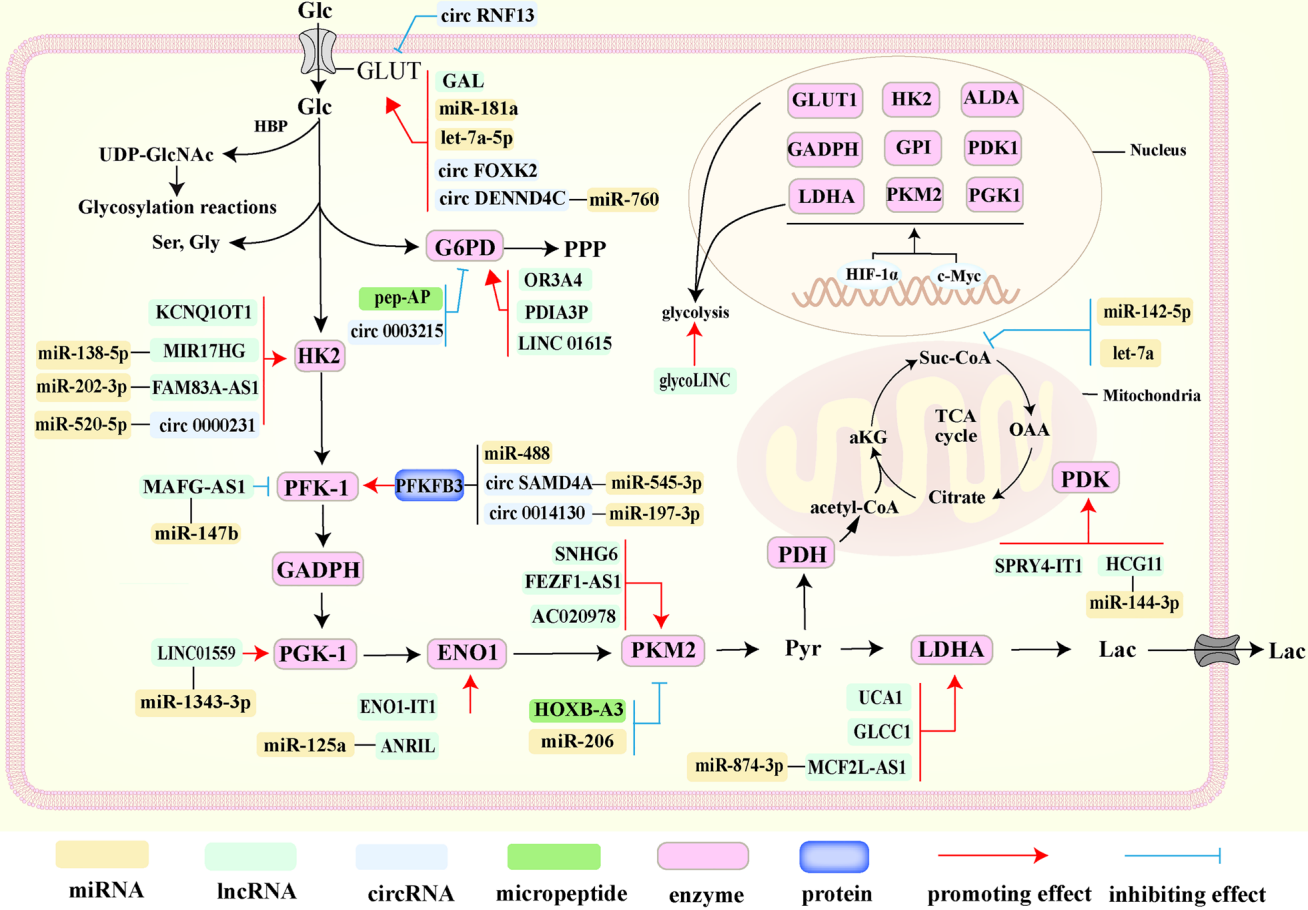
Dysregulated ncRNAs involve in glucose metabolism

It has been well-documented that abnormal glucose metabolism is one of the leading causes of cancer development [63]. Usually, normal tissues acquire energy through aerobic oxidative phosphorylation but undergo anaerobic glycolysis under hypoxia. However, the main way to acquire energy in cancer cells is aerobic glycolysis even under the condition of adequate oxygen. Accumulated evidence suggests there are numerous glucose metabolism-associated ncRNAs dysregulated in cancers resulting in mitochondrial dysfunction, abundant activation of key enzymes, altered isozyme profiles and dysregulated glucose metabolic signalling pathways [64–66]. Current progress in the role of ncRNAs in glucose metabolism has been summarized in Fig. 1.

#### NcRNAs and glucose uptake

GLUT is a key transporter protein for glucose uptake. Extensive research has demonstrated that dysregulation of ncRNAs in cancer mediates aberrant expression of GLUT1, such as miR-181a and let-7a-5p. The increased expression level of miR-181a promotes metastasis in CRC by suppressing phosphatase and tensin homolog (PTEN) expression and promoting GLUT1 expression [67]. In contrast, the low expression level of let-7a-5p in triple-negative breast cancer (TNBC) patients was negatively correlated with GLUT12 and promoted the proliferate, migrate, and invade ability of TNBC cells [68]. It is suggested that circRNF13 plays an essential role in the glucose metabolism programming of nasopharyngeal carcinoma (NPC) by stabling SUMO2 mRNA, increasing the SUMO and ubiquitination degradation of GLUT1 [69]. Li et al. found that increased expression of lncRNA GAL promotes colorectal cancer (CRC) liver metastasis. Mechanically, GAL interacts with GLUT1 to increase the SUMOylation of GLUT1 and thereby promote the stability of its mRNA [70]. Moreover, studies have revealed that circDENND4C promotes GLUT1 expression by sponging miR-760 contributing to the rapid proliferation of CRC [71]. Cui and his colleagues have illustrated that m6A modification of circFOXK2 promotes stability of GLUT1, which in turn plays a carcinogenic role in oral squamous cell carcinoma [72]. Above all, dysregulated ncRNAs affect GLUT-mediated glucose uptake rate in cancer cells related to malignant progression.

#### NcRNAs and glycolysis

Hexokinase (HK) is the first rate-limiting enzyme in glycolysis. The increased expression of HK2 in cancer is positively correlated with cancer malignancy, which is regulated by numerous ncRNAs. A recent study has revealed that increased expression of lncRNA KCNQ1OT1 in CRC promotes proliferation by decreasing the ubiquitination level of HK2, resulting in increased aerobic glycolysis [73]. LncRNA MIR17HG promotes liver metastasis through miR-138-5p/HK1 axis, which induces glycolysis. Intriguingly, the product of glycolysis, lactate, can facilitate the transcription of MIR17HG by activating the p38/Elk-1 signaling pathway, forming a positive feedback loop [74]. Another study related to lung adenocarcinoma has suggested that upregulation of lncRNA FAM83A-AS1 promotes the expression of HK2 through sponging miR-202-3p, leading to increased glycolysis [75]. A similar effect of circ0000231 on aerobic glycolysis in CRC cells has been illustrated in the research by Liu et al*.* [76]. According to the latest findings above, by targeting the lncRNA-miRNA-mRNA competitive endogenous RNA (ceRNA) regulatory network, which modulates HK expression could be a promising strategy to inhibit cancer malignant progression.

Phosphofructokinase-1 is one of the key enzymes in the glycolytic process. Cui et al*.* has reported that upregulated expression of lncRNA MAFG-AS1 in CRC promotes cancer proliferation, cell cycle progression, and inhibits cell apoptosis by targeting the miR-147b/NDUFA4 axis to upregulate phosphofructokinase-1 (PFK-1) expression [77]. 6-phosphofructo-2-kinase/fructose-2,6-bisphosphatase 3 (PFKFB3), encoded by the pfkfb3 gene, is abundantly expressed and closely related with the generation of chemotherapy resistance. There is sufficient evidence showing that dysregulated ncRNAs are involved in regulating the expression of PFKFB3. Deng et al*.* have demonstrated that increased expression of miR-488 in CRC can inhibit glucose uptake and lactate secretion by targeting PFKFB3 and improve CRC sensitivity to oxaliplatin/5-Fu [78]. In addition, the latest identified circSAMD4A and circ0014130, which are highly expressed in CRC, contribute to the 5-Fluorouracil (5-Fu) resistance by acting as ceRNAs resulting in the increased expression of PFKBF3 [79, 80]. In conclusion, there is sufficient evidence showing that PFKBF3 dysregulation mediated by ncRNAs is closely associated with chemoresistance in cancers. Importantly, targeting ncRNAs may be an effective strategy to overcome chemotherapy resistance caused by high expression of PFKFB3.

Recent studies have reported that ncRNAs generated from intestinal microbial are involved in cancer development [81, 82]. *F.nucleatum* promotes the transcription factor SP1 to bind with the promoter region of lncRNA ENO1-IT1 to activate ENO1-IT1 transcription causing increased glucose metabolism in CRC to further support carcinogenesis. It suggests a complicated interaction between ncRNAs deriving from microbiome and glucose metabolism [83]. Besides, alpha-enolase (ENO1) has been identified by Ma et al*.* acts as the target of lncRNA ANRIL/miR-125a pathway inducing drug resistance in TNBC cells [84]. Wang et al. found that mesenchymal stem cells (MSCs) promoted cancer progression by transporting exosomes containing LINC01559 to gastric cancer cells. Increased expression of LINC01559 activates the PIK3/AKT pathway by sponging miR-1343-3p to upregulate phosphoglycerate kinase 1 (PGK1). In addition, LINC01559 induces methylation of the PTEN promoter, which triggers the PIK3/AKT signaling pathway by inhibiting PTEN and accelerates cancer progression [85].

Pyruvate kinase is another key enzyme in glycolysis. Recent research has reported that miR-206 and lncRNA SNHG6 play oncogenic and pro-cancer roles in CRC by targeting heterogeneous ribonucleic acid proteins to alter pyruvate kinase isozyme gene splicing patterns and control the PKM1/PKM2 ratio, respectively [86, 87]. The small peptide HOXB-A3 encoded by lncRNA HOXB-AS3 is revealed to inhibit the splicing formation of pyruvate kinase M2 (PKM2) by competitively binding with the arginine residue in the hnRNP A1 RGG motif to exert cancer suppressive effects [88]. LncRNA FEZF1-AS1 enhances the stability of PKM2 by binding to its A2 structural domain, leading to elevated PKM2 levels in the cytoplasm and nucleus. FEZF1-AS1 induces increased PKM2 in the cytoplasm to promote aerobic glycolysis, and in the nucleus to further activate the signal transducer and activator of transcription 3 (STAT3) signaling pathway [89]. Hua et al*.* have shown that upregulation of lncRNA AC020978 is transcriptionally activated by HIF-1α in non-small cell lung cancer, which interacts with PKM2 and enhances the stability of PKM2 protein. In addition, AC020978 can also increase the interaction of PKM2 with HIF-1α by promoting the nuclear translocation of PKM2, further enhancing the transcriptional activation of HIF-1α on target genes [90]. NcRNAs regulate the Warburg effect by alternatively splicing pyruvate kinase isozymes and controlling the ratio of PKM1/PKM2. Targeting ncRNAs to regulate the splicing of PKM may be an effective strategy to suppress aerobic glycolysis.

Apart from the above-mentioned key enzymes and transporters in glycolysis, lactic dehydrogenase is a critical rate-limiting enzyme for glycolysis. It is identified that lncRNA MCF2L-AS1 promotes the expression of heat shock protein 90 (HSP90) by sponging miR-874-3p in CRC cells leading to the increased production of lactic acid [91]. Tang et al*.* found that lncRNA GLCC1 reduces ubiquitination of c-Myc by directly interacting with HSP90 to modify the transcriptional pattern of the target gene lactate dehydrogenase A (LDHA), thereby reprogramming glucose metabolism to promote CRC progression [92]. Interestingly, it is reported that a c-Myc-regulated lncRNA glycoLINC can act as a backbone to assemble several glycolysis-related enzymes into a metabolon that exerts a pro-carcinogenic effect by increasing the catalytic efficiency of glycolytic enzymes leading to an increase in glycolytic flux [93]. The increased expression of LDHA is regulated by many oncogenes, promoting glycolysis and malignant progression of cancers, which is mediated by ncRNAs. Thus, targeting dysregulated ncRNAs may be potential targets to suppress cancer development through regulating the expression of LDHA.

#### NcRNAs and the pentose phosphate pathway

The PPP is an important metabolic bypass for glucose and glucose-6-phosphate dehydrogenase (G6PD) is the key rate-limiting enzyme. A recently published study has suggested that LINC01615 in CRC increased pentose phosphate pathway (PPP) flux, reduced reactive oxygen species (ROS) production, and promoted nucleotide and lipid synthesis by regulating the splicing of G6PD precursor mRNA [94]. Chen et al*.* discovered that circ0003215 suppressed PPP and the malignant phenotype of CRC through the miR-663b/ DLG4 axis. Mechanistically, DLG4, which is mediated by circ0003215 and upregulated by K48-linked G6PD, increased its ubiquitinated degradation and inhibited the PPP [95]. In osteosarcoma, lncRNA OR3A4 increases G6PD expression through sponging miR-1207-5P, leading to the activation of PPP, thus promoting cancer cell growth [96]. In addition, the short peptide pep-AP encoded by lncRNA AP has been validated to facilitate oxaliplatin by interacting with TALDO1, decreasing NADPH/NADP^+^ and glutathione (GSH) levels [97]. Yang et al. revealed that high expression of lncRNA PDIA3P in multiple myeloma binds to c-Myc to increase its transcriptional activity and promotes c-Myc interacting with the G6PD promoter, leading to increased G6PD expression and malignant progression [98]. In a word, ncRNAs play a critical role in sustaining nucleotide biosynthesis as well as maintaining intracellular redox homeostasis through the PPP pathway. However, the PPP pathway mediated by dysregulated ncRNAs can provide numerous synthetic raw materials for cancer proliferation, while blocking this can be a promising strategy for cancer treatment.

#### NcRNAs and the tricarboxylic acid cycle

Mitochondria are vital for the complete oxidative phosphorylation of glucose. After glucose is converted to pyruvate via the glycolytic route, the pyruvate dehydrogenase complex (PDC) in the mitochondria catalyzes the formation of acetyl CoA, which enters the tricarboxylic acid cycle (TAC). In malignancies, abnormal activation of pyruvate dehydrogenase kinases (PDKs), inhibitors of pyruvate dehydrogenase, leads to increased glycolytic flow. PDC activity is inhibited in cancer cells by phosphorylation of the E1a subunit of PDKs, restricting pyruvate entrance into mitochondria [99].

In CRC, an lncRNA HCG11/ miR-144-3p/ PDK4 axis has been illustrated to promote aerobic glycolysis and lactate generation [100]. LncRNA SPRY4-IT1 is documented to bind to PDK1 and boost its stable expression, resulting in enhanced aerobic glycolysis and accelerated CRC progression [101]. Succinate dehydrogenase (SDH) in the TAC is regulated by ncRNA such as miR-142-5p, which is increased in CRC and diminishes oxygen intake while increasing lactate generation by targeting SDHB [102]. Let-7a enriched in extracellular vesicles (EVs) hampers oxidative phosphorylation of mitochondria and CRC development via the Lin28a/SDHA signaling pathway [103]. Dysregulation of enzymes such as PDK and SDH mediated by ncRNAs in cancers regulate aerobic glycolysis by limiting pyruvate access to mitochondria for complete oxidative phosphorylation. It may play a key role in cancer treatment in the future.

Defective mitochondrial function and abnormal biogenesis are often also important factors in promoting aerobic glycolysis, which is regulated by nuclear respiratory factors 1 and 2 (NRF1 and NRF2), mitochondrial transcription factor A (TFAM) and ncRNAs [104]. According to a recently released study, LINC00467 encodes an ATP synthase-associated peptide (ASAP), which interacts with the ATP5A and ATP5C to increase ATP synthase activity and mitochondrial oxygen consumption rate [105]. Lnc 00839 functions as a scaffold in the nucleus, enhancing the transcriptional activity of NRF1, which has a pro-carcinogenic impact on CRC by promoting oxidative phosphorylation (OXPHOS) and mitochondrial formation [47]. Reduced ROS and NAD^+^/NADH have been reported as a result of miR-483, miR-676, and miR-877 downregulating the expression of mitochondrial metabolism-related genes [106]. According to Zhao et al., the nuclear-encoded lncRNA MALAT1, aberrantly enriched in hepatocellular carcinoma cells, promotes the synthesis of mitochondrial DNA (mtDNA) by binding to it and altering its methylation and mitochondrial function, thus controlling mitochondrial metabolism at the epigenetic level [107]. Given all this, targeting abnormal mitochondria through ncRNAs offers great possibilities for treating cancers.

### The effects of ncRNAs on cancer lipid metabolic reprogramming

**Fig. 2:**
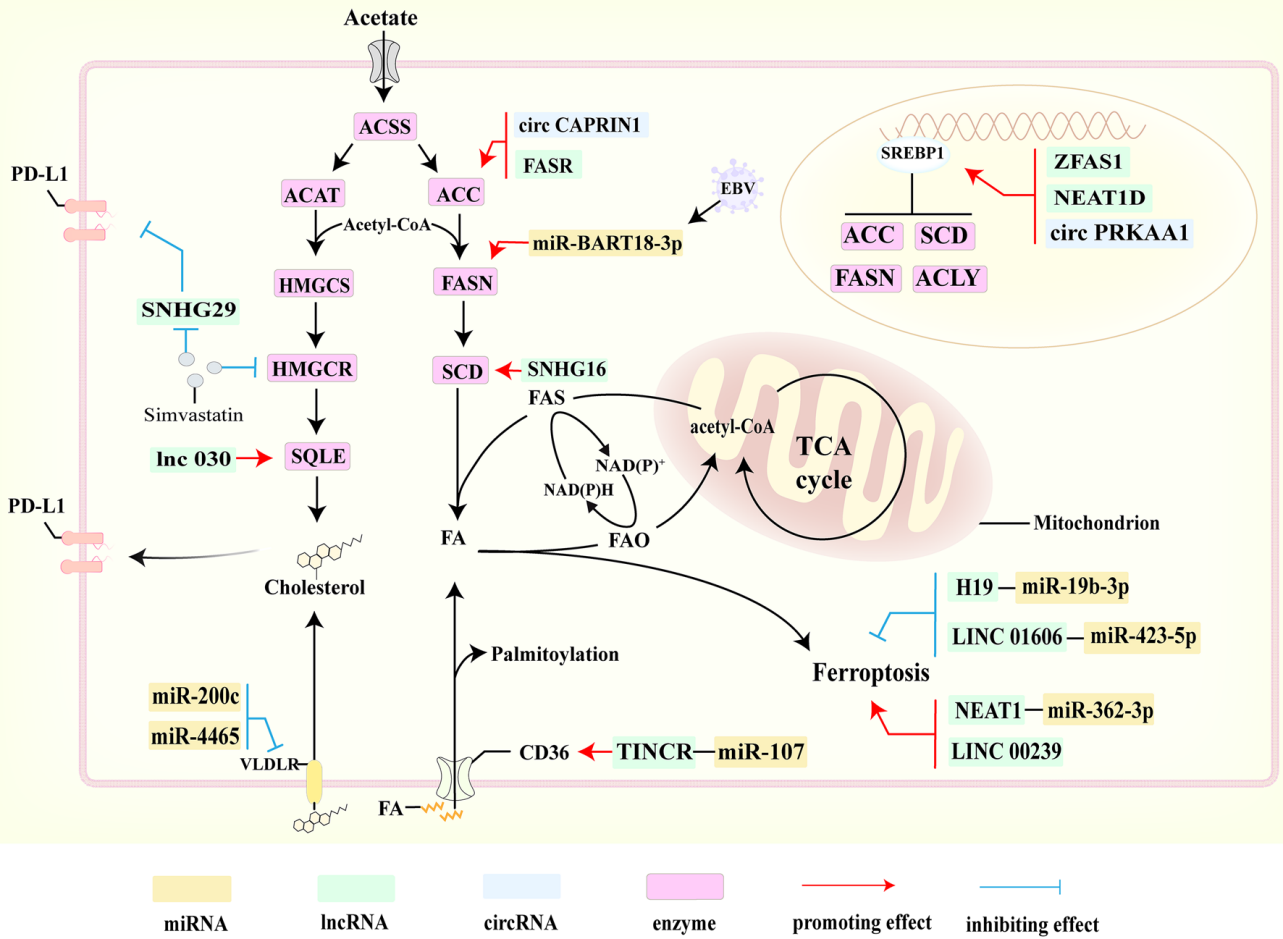
Dysregulated ncRNAs involve in lipid metabolism

Accumulated studies have demonstrated that dysregulated ncRNAs are involved in the progression of cancer lipid metabolic reprogramming including abnormal lipid uptake, accumulation, synthesis and oxidation *et.al*. [108–112]. The role of ncRNAs in lipid metabolism has been summarized in Fig. 2.

#### NcRNAs and lipid uptake and accumulation

The uptake of exogenous fatty acids is mainly dependent on the transmembrane transport of CD36, fatty acid transport proteins (FATPs) and fatty acid binding proteins (FABPs). In contrast, cellular uptake and utilization of cholesterol are dependent on the low-density lipoprotein receptor superfamily (LDLRs). It has been reported that miR-107 overexpressed in CRC cells decreases CD36 expression by inhibiting the PPAR signaling pathway. In contrast, lncRNA TINCR, an endogenous competing RNA for miR-107, inhibits its expression and activates CD36 to play a role in inhibiting cell proliferation and promoting apoptosis [110].

The decreased expression of very low-density-lipoprotein receptor (VLDLR) in cancer cell membranes contributes to the accumulation of cholesterol in the cytoplasm. It has been found that VLDLR downregulation promotes CRC cell proliferation and migration through elevated miR-200c expression [113]. Mosapour et al. found that miR-4465 targets VLDLR to promote the development of breast cancer, resulting in cholesterol accumulated in cytoplasm [114]. Abnormally high cholesterol in hepatocellular carcinoma cells promotes malignant lesions in non-alcoholic fatty liver disease (NAFLD) by inducing lncRNA SNHG6 to localize in the endoplasmic reticulum-lysosome interactions region and activating the mammalian target of rapamycin (mTORC1) signaling pathway [115]. In contrast, LncRNA Mexis and LINC DYNLRB2-2 induce macrophage ABCA1 gene expression, which in turn promotes ABCA1-mediated cholesterol efflux leading to increased cholesterol levels in TME, which may further promote cholesterol uptake by cancer cells [116, 117]. Above all, high lipid accumulation in cancer cells promotes the development of cancer. Therefore, limiting lipid uptake and abnormal intracellular lipid accumulation through ncRNAs may be a promising strategy to improve cancer treatment efficiency.

#### NcRNAs and lipid synthesis

It has been shown that lncRNA SNHG16 targets various lipid metabolizing enzymes such as stearoyl-CoA desaturase (SCD) and ATP-citrate synthase (ACLY) through endogenous competing RNAs in CRC. And high expression of SNHG16 positively correlates with the expression of transcription factors long-chain-fatty-acid-CoA ligase 2 (ASCL2), ETS2 and c-Myc, which are activated by the Wnt signaling pathway [111]. Acetyl coenzyme A carboxylase (ACC), a rate-limiting enzyme for fatty acid synthesis, has been found transcriptional activation by circCAPRIN1 in CRC through direct binding to STAT2, causing increased lipid synthesis and CRC progression [118]. Peng et al. demonstrated that LncRNA FASR expression was driven by USF1 via a super-enhancer. FASRL enhances fatty acid synthesis and lipid accumulation by binding to the fatty acid synthesis rate-limiting enzyme acetyl coenzyme A carboxylase (ACAC), thereby promoting hepatocellular carcinoma (HCC) [119]. A study has revealed that miR-BART18-3p, encoded by EBV, activates the HIF-1α/LDHA axis by targeting sirtuin to promote lactate accumulation and acetyl coenzyme A production under hypoxic conditions, resulting in enhanced acetylation of histones H3K9, H3K14 and H3K27 of fatty acid synthase (FASN) and activates de novo synthesis of fat [120]. Accumulated research has proved that dysregulated ncRNAs play an indispensable role in regulating lipid metabolism.

The mevalonate pathway is a key signaling pathway for cholesterol synthesis, which can not only be activated by MYC but also lead to up-regulation of MYC expression through up-regulation of miR-33b, forming a positive feedback loop [121]. MiR-195 has been illustrated that effectively prevent the accumulation of cholesterol by inhibiting the expression of IKKα and TAB3 [122]. A previous study has revealed that lncRNA 030 maintains the stemness of breast cancer stem cells by interacting with PCBP2, regulating the stability of squalene monooxygenase (SQLE) mRNA, promoting cellular cholesterol synthesis, and further leading to activation of the PI3K/AKT pathway [123]. Ni et al. revealed that an HMG-CoA reductase (HMGCR) reductase inhibitor, simvastatin, could promote anticancer effects by inhibiting lncRNA SNHG29-mediated Yes-Associated Protein (YAP) activation and suppressing PD-L1 expression [124]. It has been demonstrated that excessive accumulation of cholesterol induces PD-L1 expression in cancer cells [5]. Therefore, inhibition of cancer cell cholesterol synthesis pathway by targeting ncRNAs may be an emerging direction to improve the efficacy of immunotherapy. Furthermore, Wang et al. found that lncRNA ZFAS1 increased the expression level of SCD1 and FASN, through bound PABP2 to stabilize the mRNA stability of SREBP1, leading to promoting lipid accumulation in CRC [125]. Highly expressed RPRD1B in gastric cancer cells increases fatty acid uptake and synthesis through transcriptional upregulation of c-Jun/c-Fos and activation of the c-Jun/c-Fos/SREBP1 axis [126]. Liu et al. have shown that upregulated circPRKAA1 can form a tetrameric complex with Ku80/Ku70 heterodimer and mSREBP-1 increasing the de novo synthesis of lipids through transcriptional activation of ACC1, ACLY, SCD1 and FASN [127]. Generally, increased lipid synthesis is tightly related to cancer progression, which can be induced by dysregulated ncRNAs through targeting lipid-related enzymes and their key components SREBPs.

#### NcRNAs and lipid oxidation

It has been demonstrated by Gharib et al. that miR-497-5p plays an oncogenic role in CRC by targeting ACSL5 to block the oxidation activation of fatty acids [112]. Barisciano et al. research suggests that miR-27a may act by targeting carnitine palmitoyltransferase-1 (CPT-1) and acyl coenzyme a dehydrogenase-9 (ACAD9) in the process of fatty acid β-oxidation. PPARγ coactivator-1α (PGC-1α), which directly regulates peroxisome proliferators-activated receptors (PPARγ), is increased in CRC cells when miR-27a is knocked down. Also, protein levels of the downstream effectors of PPARγ, CPT1A and ACAD9, are upregulated [128]. Li et al. showed that in the condition of serum deprivation, highly expressed circRNAACC1 acted as a cancer-promoting factor to control fatty acid β oxidation of CRC by activating AMP-activated protein kinase (AMPK) [129].

Ferroptosis, which develops from an increase in iron-dependent lipid peroxide, is regulated by a network of ncRNAs [130, 131]. According to Luo et al., LINC01606 serves as the ceRNA of miR-423-5p to upregulate SCD1 and further stimulate the Wnt/β-catenin signaling pathway, which prevents ferroptosis in CRC cells and preserves cell stemness [132]. Zhang et al. revealed that the ferroptosis inducers Erastin and RSL3 promoted lncRNA NEAT1 expression by facilitating p53 binding to its promoter, increasing intracellular ROS production [133]. It has been reported that LINC00239 resists ferroptosis by interacting with the Kelch structural domain of Keap1, thereby enhancing Nrf2 protein stability promoting its nuclear translocation and further reducing the activity of Erastin and RSL3 [134]. Zhang et al. reported that curcuminol can induce ferroptosis in lung cancer cells through lncRNA H19/miR-19b-3p/FTH1 axis [135]. Collectively, cell death plays a significant role in several biological processes and the targeting of cell death emerges as a prominent therapeutic strategy for the treatment of a variety of diseases, especially cancers [131]. Ferroptosis, an emerging type of cell death, is distinct from apoptosis and necrosis in the conventional sense [131]. Thus, revealing the molecular mechanisms of lncRNAs mediated-ferroptosis could provide new insights into the cell death processes and potential molecular components to develop promising strategies to prevent and stimulate cell death.

### The effects of ncRNAs on amino acid metabolic reprogramming

**Fig. 3:**
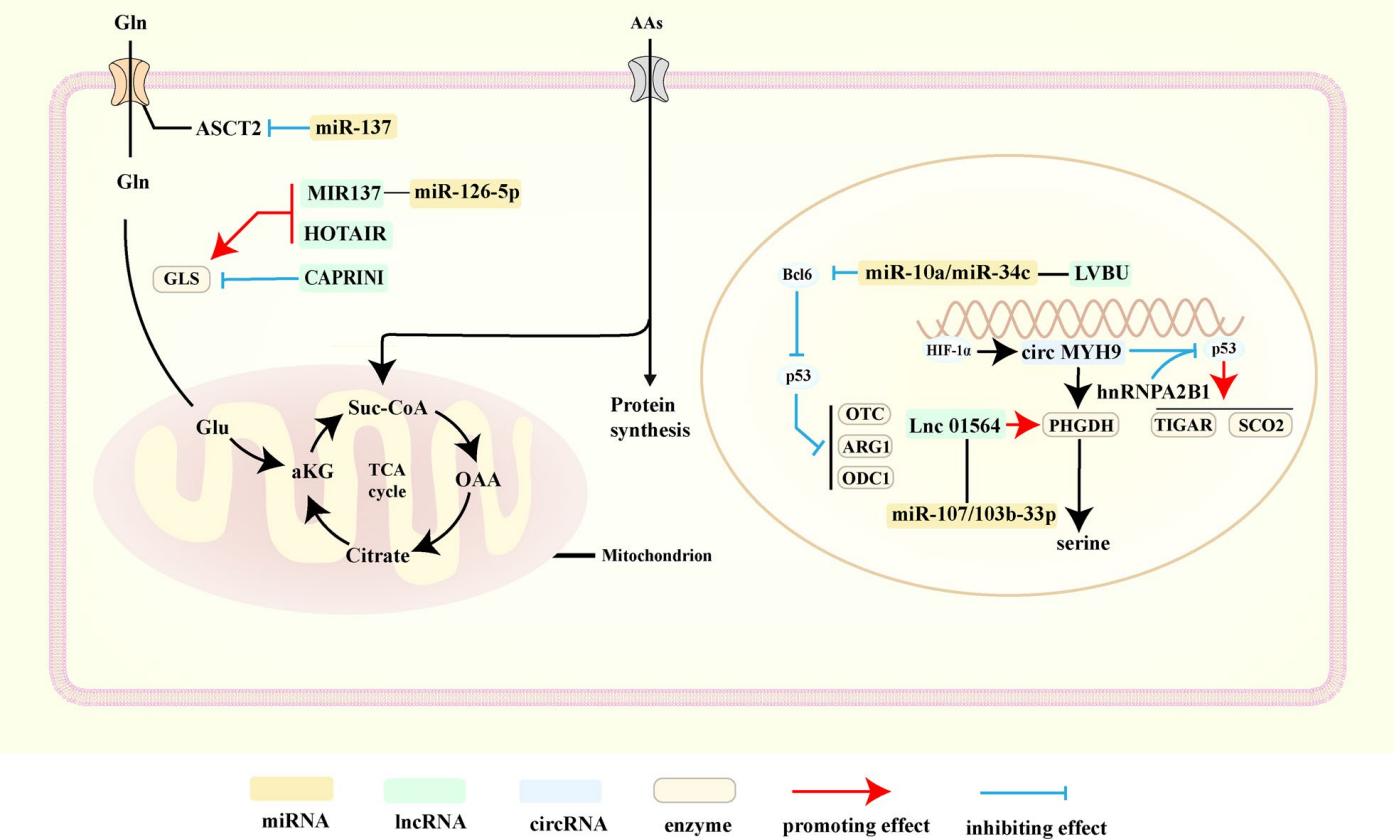
Dysregulated ncRNAs involve in amino acid metabolism

Emerging studies have revealed that altered amino acid metabolism is closely associated with cancer outgrowth, metastasis, and therapeutic resistance [136, 137]. On another hand, numerous studies have shown that dysregulated ncRNAs are involved in regulating the amino acid metabolic reprogramming, which facilitates cancer progression [138, 139]. The latest progress in the role of ncRNAs in amino acid metabolism has been summarized in Fig. 3.

#### NcRNAs and glutamine

Rapidly proliferating cancer cells often exhibit an extremely strong dependence on glutamine [140]. It is Dong et al. show that miR-137 directly regulates amino-acid transporter 2 (ASCT2) expression, which significantly reduces CRC cells glutamine uptake [141].

Glutaminase (GLS) is an amino acid enzyme that has been identified as a target of miR-137. Heat shock factor 1 (HSF1) can inhibit the interaction of miR-137 and GLS to promote glutamine catabolism and activate mTOR signaling, which contributes to cancer development [138]. Liu et al*.* have reported that lncRNA HOX-AS1 can increase the expression of GLS and the levels of intracellular glutamine metabolism through sponging miR-126-5p [139]. Moreover, the latest research has demonstrated that lncRNA CAPRIN1 can inhibit GLS1 translation by driving CAPRIN1-mediated phase segregation in glutamine deficiency, preventing excessive depletion of limited glutamine to maintain cancer survival [142]. Wang et al*.* have demonstrated that downregulated miR-375 significantly promotes CRC development by activating the PI3K/Akt pathway and increasing the expression of glutamic–pyruvic transaminase (GPT2) [143, 144]. Limiting cancer cells to uptake glutamine can be an effective strategy to inhibit cancer growth, while ncRNAs play an important role in regulating glutamine metabolism. Therefore, targeting ncRNAs associated with glutamine metabolism is likely to be a potential approach to limit cancer progression.

#### NcRNAs and other amino acids

Apart from glutamine, there are numerous research revealed the potential role of ncRNAs in other amino acid metabolism.

Serine and glycine play an essential role in maintaining the anabolic requirements of cancer and supporting proliferation by controlling redox states [145]. Zhu et al. demonstrated that gLINC increased glycolytic flux under normal conditions and promoted cancer cell survival in serine deficiency [93]. Zhang G et al. has demonstrated that LncRNA 01564 is upregulated in HCC, which promotes the survival of HCC under glucose deprivation by targeting miR-107/103b-3p/ PHGDH axis [146]. According to Liu *et.al*, increased expression of circMYH9 binds the m6A reader hnRNPA2B1 into the nucleus, which reduces the stability of p53 pre-mRNA, while increasing endogenous serine production through enhancing phosphoglycerate dehydrogenase (PHGDH) activity. Amino acid deficiency leads to greater cellular production of ROS, which can enhance circMYH9 expression by attaching to the promoter region of circMYH9 and increasing endogenous serine production by increasing the expression of HIF-1α [147].

NcRNAs can also target metabolic pathways related to amino acids to exert regulatory effects. In esophageal squamous cell carcinoma, the increased LncRNA SLC25AS1-AS can not only maintain the stability of SLC25A21 mRNA but also reduce the intracellular NAD^+^/NADH ratio by affecting tryptophan catabolism [148]. Ornithine is the initiator of polyamine synthesis, increased expression of polyamine synthesis genes in cancer promotes cancer progression [149].

Ornithine decarboxylase 1 (ODC1), a crucial enzyme for polyamine biosynthesis using ornithine from the urea cycle, is frequently deregulated in cancer [150]. According to Meng et al. hypoxia-induced lncRNA LVBU accelerates CRC cell proliferation by regulating the BCL6-p53 axis and urea cycle/polyamine production pathway. LVBU overexpression can reduce the degradation of B-cell lymphoma 6 protein** (**BCL6) by sponging miR-10a/miR-34c, which deregulates p53 on arginase1, ornithine transaminase and ODC1 genes and further accelerates CRC cell proliferation [151].

According to a previous study, the high expression of LINC01234 encourages HCC proliferation and migration by interacting with the promoter of Argininosuccinate Synthase 1 (ASS1) and blocking p53-mediated transcriptional activation, which raises aspartate levels and activates the mTOR signaling pathway [152]. Li et al. has found that HBXIP protein interacts with c-Myc through the leucine zipper, recruiting lncRNA Hotair and histone demethylase LSD1 to transcriptionally activate c-Myc. Silencing Hotair blocked the growth-promoting effect of c-Myc in breast cancer cells [153]. Ma et al. has discovered that LINC01431 increases the interaction between PRMT1 and covalently closed loop DNA (cccDNA) of hepatitis B virus (HBV) to further limit HBVs replication by boosting H4R3me2a modification on cccRNA. The viral protein HBx maintains persistent HBV replication by escaping LINC01431-mediated transcriptional repression through the transcription factor ZHX2, which inhibits transcription of the host LINC01431 [154]. In addition, Yan et al. has shown that SRSP, encoded by lncRNA LOC90024, can bind with serine and arginine-rich splicing factor 3 (SRSF3) to regulate mRNA splicing [155]. In conclusion, amino acid metabolism regulated by ncRNAs has suggested novel targets for cancer therapy in the future. However, are more studies needed to figure out the potential mechanism of amino acid metabolic reprogramming mediated by dysregulated ncRNAs.

## NcRNAs effect cancer therapeutic tolerance through metabolic reprogramming

Chemotherapy, radiation, targeted therapy and immunotherapy are the major strategies used for malignant cancers. However, the outcome of patients does not remain satisfactory due to limitations such as multi-drug resistance and tumor relapse [156]. Numerous types of research have implicated that ncRNAs may be a potential target to overcome therapeutic tolerance. Nevertheless, the potential regulatory mechanism for the occurrence of therapeutic tolerance and metabolic reprogramming in cancer needs to be further investigated.

### NcRNAs mediate chemotherapy resistance through metabolic reprogramming

**Fig. 4:**
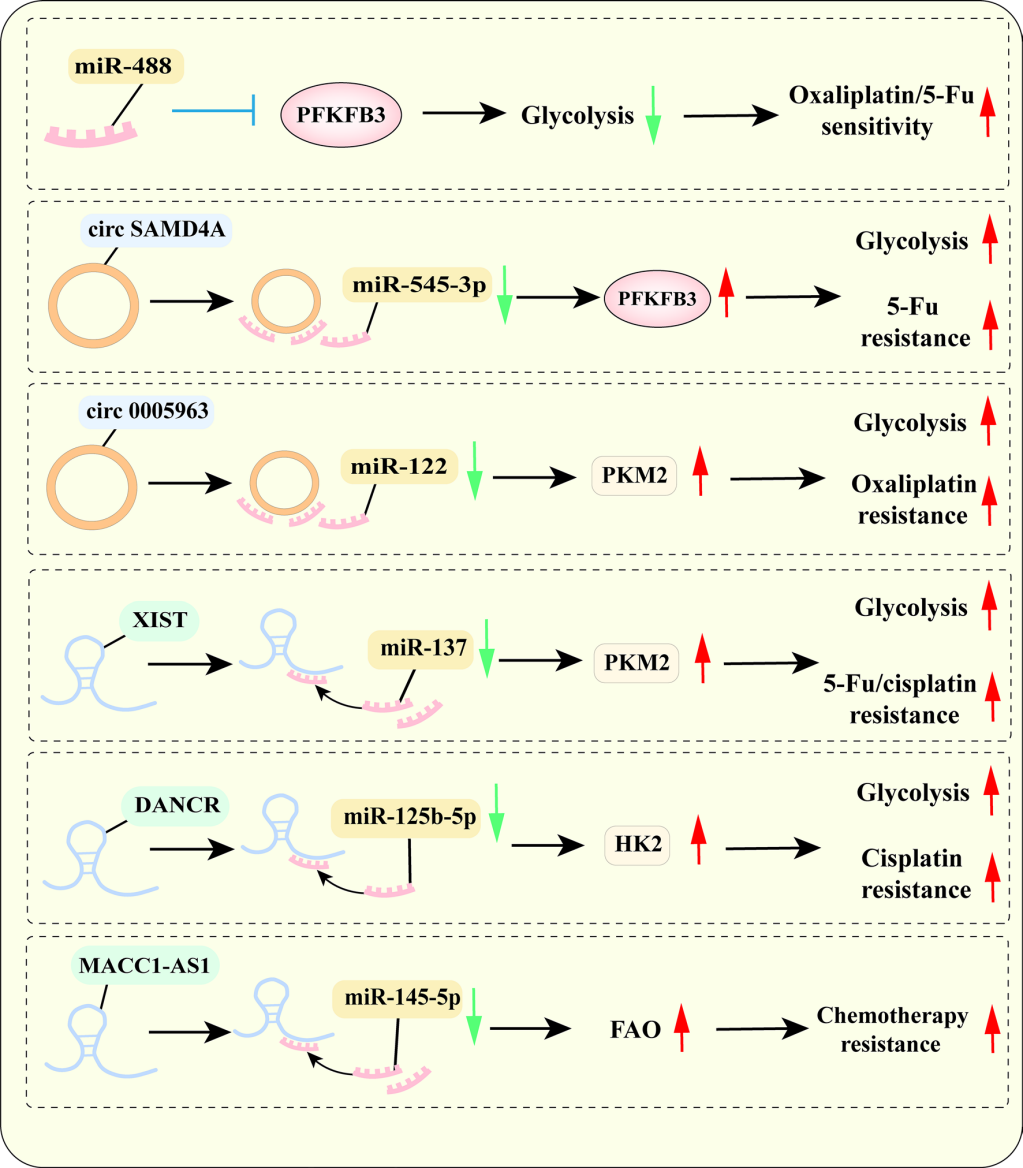
The mechanism of ncRNAs mediate chemotherapy resistance through metabolic reprogramming

Accumulated studies have demonstrated that ncRNA-mediated metabolic reprogramming is involved in regulating the chemotherapy resistance and it has been summarized in Fig. 4 [7]. MiR-488 has been found to promote oxaliplatin and 5-Fu-induced CRC cell apoptosis and inhibit glucose metabolism by targeting PFKFB3 [78]. In addition, it has been illustrated that circSAMD4A and circ0014130 can significantly promote 5-Fu resistance in CRC cells by acting as miRNA sponges to upregulate PFKFB3 expression and lactate generation [79, 80]. The study by Huang et al. has shown evidence that transport circ0005963 deriving from oxaliplatin-resistant cells to susceptible cells can promote oxaliplatin resistance by sponging miR-122 to activate PKM2 [157]. Apart from circ0005963, lncRNA XIST is also implicated in sponge miR-137 to enhance the resistance of 5-Fu/cisplatin in CRC cells by maintaining a higher PKM2/PKM1 ratio [158]. HK2 is an essential enzyme for glycolysis, which has been elaborated to be targeted by the LncRNA DANCR/miR-125b-5p axis involved in regulating the glucose metabolism of CRC cells and inducing cisplatin resistance [159].

A similar effect of ncRNAs on other metabolism reprogramming has been illustrated in vast research. It is demonstrated that increased expression of lncRNA MACC1-AS1 is documented to promote 5-Fu and oxaliplatin resistance by sponging miR-145-5p to inhibit the fatty acid oxidation pathway [160]. Furthermore, increased expression of miR-1291 is shown to target GLUT1-mediated glycolysis and ASS1-mediated arginine hydrolysis pathways, enhancing the susceptibility of prostate cancer (PC) cells to arginine deprivation and cisplatin therapy [161]. Li et al. have identified that re-expression of miR-34a in oxaliplatin-resistant CRC cells can increase their mRNA stability and restore sensitivity to oxaliplatin by targeting ornithine decarboxylase antizyme 2 (OAZ2) [162]. NcRNA involved in regulating metabolic reprogramming and chemoresistance will serve as novel anticancer strategies for cancer in the future. Nevertheless, the potential regulatory mechanism of ncRNAs mediating chemoresistance and metabolism balance needs to be further investigated.

### NcRNAs mediate radiotherapy resistance through metabolic reprogramming

**Fig. 5:**
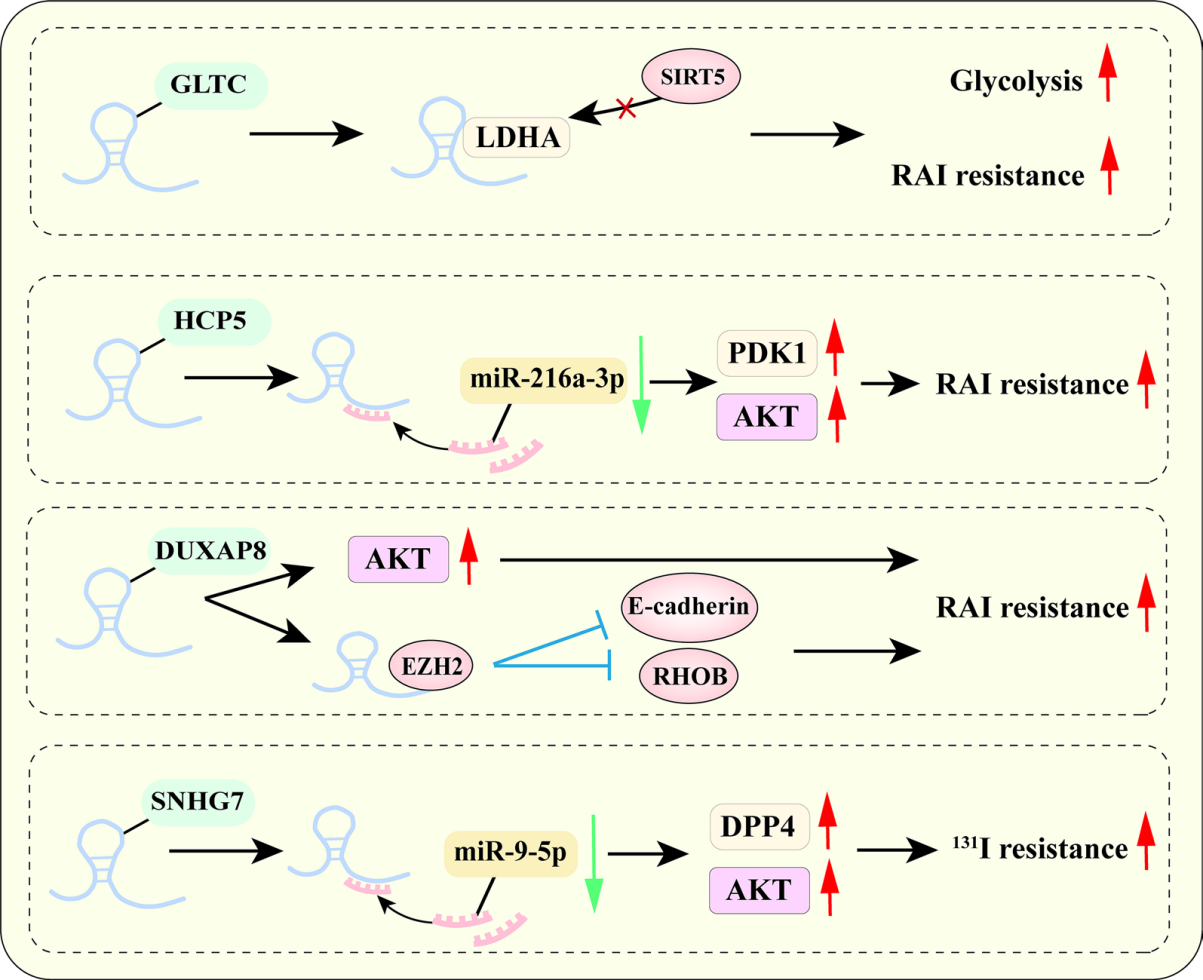
The mechanism of ncRNAs mediate radiotherapy resistance through metabolic reprogramming

Dysregulate ncRNAs facilitate radiotherapy resistance by affecting metabolic reprogramming has been summarized in Fig. 5. Liang et al. have identified that lncRNA GLTC can promote radioiodine (RAI) resistance in papillary thyroid cancer by promoting LDHA enzymatic activity [163]. Increasing research has found that the radiotherapy resistance mediated by ncRNAs is associated with the AKT pathway. For instance, increased expression of lncRNA HCP5 has been identified by Guo et al. to induce radiotherapy resistance in esophageal cancer tissues by promoting AKT activation through regulating miR-216a-3p/PDK1 axis [164]. Another well-documented lncRNA DUXAP8 increases radiotherapy resistance in breast cancer cells by activating the PI3K/AKT/mTOR signaling pathway [165]. Moreover, the expression of SNHG7 is demonstrated to be upregulated in TC tissues and correlated with [131]I resistance as the increased expression of SNHG7 promotes [131]I resistance in TC cells by sponging miR-9-5p and increasing DPP4 expression to activate PI3K/AKT [166]. Taken together, dysregulated expression of ncRNAs induces the development of radiation therapy resistance in cancer cells mainly by targeting the PI3K/AKT signaling pathway. All the above findings have provided promising ncRNAs-targeted strategies for radiotherapy resistance.

### NcRNAs mediate immune resistance through metabolic reprogramming

**Fig. 6:**
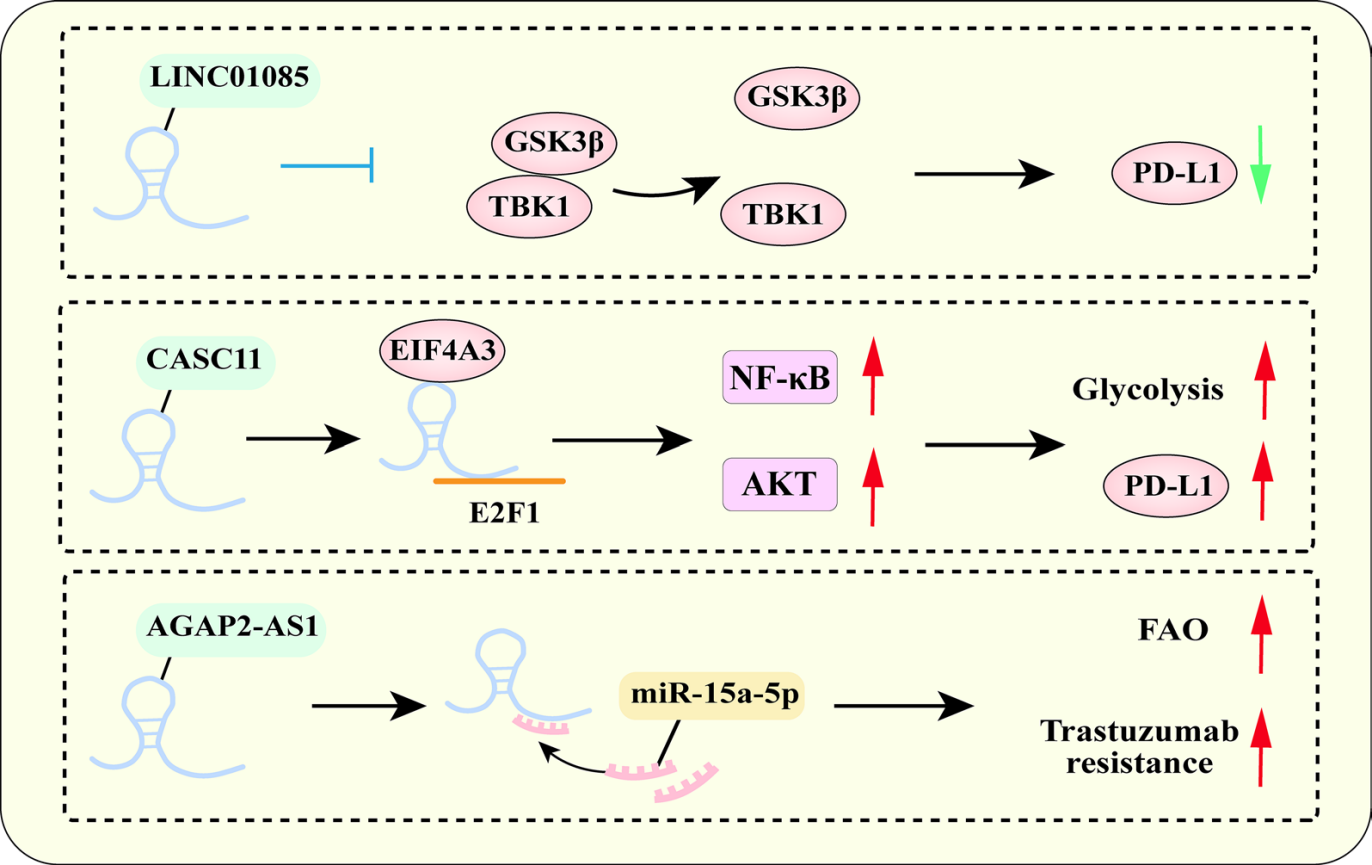
The mechanism of ncRNAs mediate immune and targeted resistance through metabolic reprogramming

Dysregulation of NcRNAs in metabolic networks is closely associated with immunotherapy tolerance and it has been summarized in Fig. 6. A currently published study has shown that overexpression of LINC01085 combined with immune checkpoint blockade is an effective strategy for the treatment of HIPC patients by inhibiting the interaction between TANK-binding kinase 1 (TBK1) and glycogen synthase kinase 3β (GSK3β) [167]. It is identified by Song *et.al* that lncRNA CASC11drives HCC progression by recruiting EIF4A3 to enhance E2F1 stability, which activates NF-κB signaling and PI3K/AKT/mTOR pathway and further upregulates glycolysis and the expression of PD-L1 [168]. As an emerging therapeutic approach, immunotherapy has been broadly applied in clinical therapy. Current findings suggest that the various combinations of treatments with produced anti-PD-1, CD80-Fc, and 4-1BBL-Fc proteins reduce inhibitory signals while increasing activatory signals, potentially lowering the risk of tumor resistance and improving treatment efficacy [169]. Based on previous studies, inhibiting NEAT1, XIST, and IL-6 may be very effective for the clinical treatment of GC [156, 170]. Recently, Elina Kaviani *et.al* revealed polyphenol natural compounds and maggot larvae (as sources for safe and effective bioactive compounds) can contribute to immunotherapy and chemotherapy activity as a complementary therapy via reducing the expression level of MKI67, CXCL8, IL-6/IL-6R, lncRNA XIST and NEAT1 and ultimately decreased the size and number of tumors [156]. Hence, the combination of ncRNAs and immunotherapy drugs is likely to be an effective strategy for improving immune treatment efficacy [167, 168, 171].

### NcRNAs mediate targeted therapy resistance through metabolic reprogramming

Dysregulation of ncRNAs mediates targeted therapy resistance through metabolic pathways has been summarized in Fig. 6. Han et al*.* have found that LncRNA AGAP2-AS1 can enhance breast cancer cell resistance to trastuzumab through miR-15a-5p/CPT1 axis to activate fatty acid oxidation (FAO) [172]. Liang et al. found that high expression of circ CDYL2 in breast cancer tissues conferred resistance to trastuzumab. Mechanically, circCDYL2 stabilizes GRB7 by preventing its ubiquitinated degradation and enhances its interaction with FAK, thereby maintaining downstream AKT and ERK1/2 activity to support trastuzumab resistance in breast cancer cells. However, HER2^+^ BC cells with high expression of circCDYL2 can reverse their resistance to trastuzumab by using FAK or GRB7 inhibitors [173]. The development of targeted therapeutic resistance is caused by dysregulated ncRNAs. To understand how metabolic reprogramming brought on by ncRNAs impacts cancer-targeted treatment, more study is required.

In conclusion, numerous studies have illustrated that dysregulated ncRNAs expression in cancer mediates the development of cancer therapeutic tolerance by regulating metabolic networks, and targeting dysregulated ncRNAs in cancer cells has great promise for overcoming cancer therapeutic tolerance, especially tolerance to chemotherapy treatment. More research is necessary to fully elucidate how ncRNAs influence cancer tolerance through metabolic reprogramming.

## Targeting ncRNAs-mediated metabolic pathways provides effective strategies for cancer therapy

There are numerous studies have demonstrated that ncRNAs are involved in multiple metabolic pathways or signaling pathways to regulate the genesis and progression of cancer cells [174–176]. Therefore, ncRNAs-based metabolic reprogramming may provide promising prospects in overcoming cancer therapeutic tolerance and improving related anti-cancer drugs’ therapeutic efficiency. Elucidating the interaction and possible mechanism between ncRNAs and metabolic reprogramming has shed some insights into understanding the pathogenesis and provided a theoretical foundation for cancer research.

**Fig. 7:**
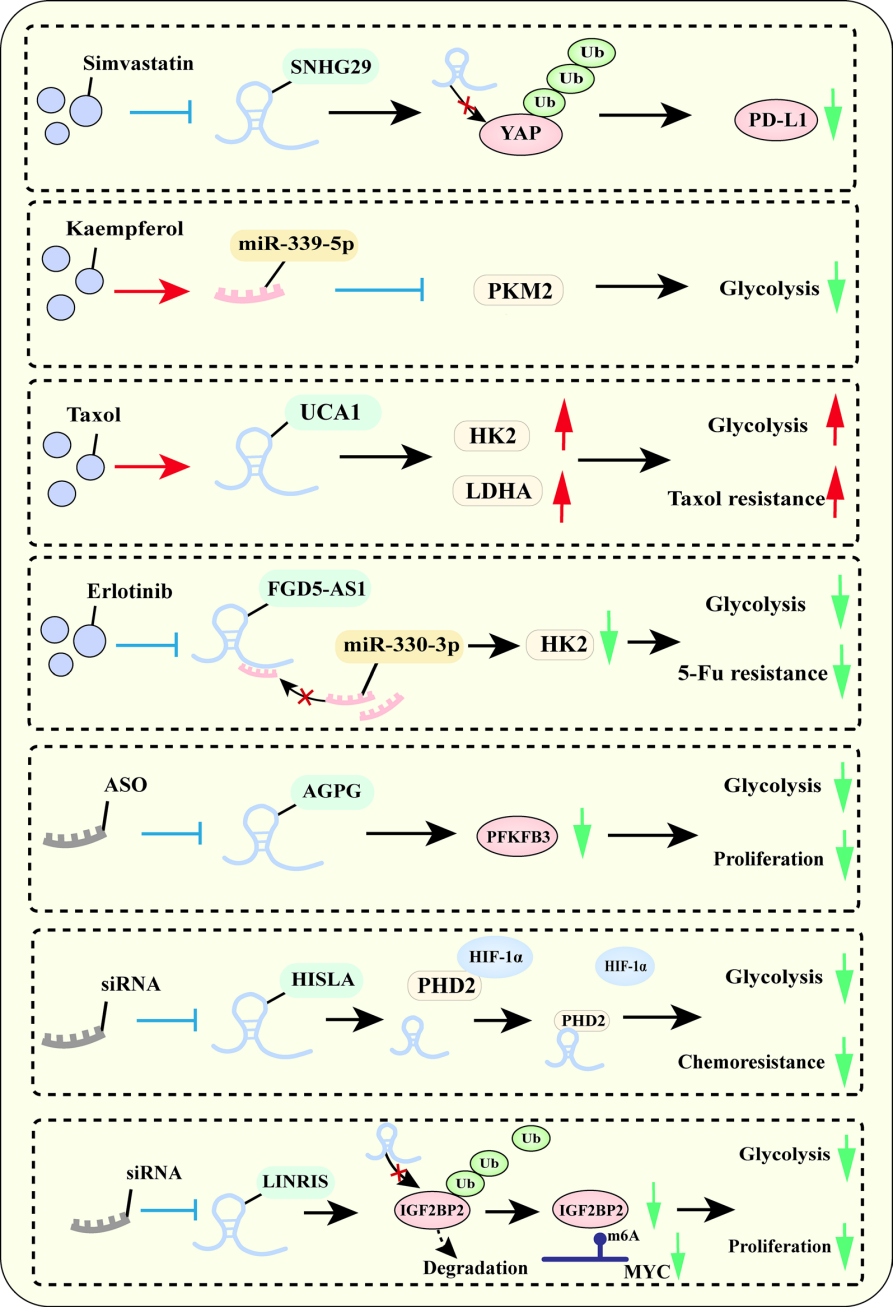
The mechanism of treatments affecting cancer progression by targeting the dysregulated ncRNAs mediated metabolic networks

The treatments suppressing cancer progression through the metabolic networks mediated ncRNAs have been summarized in Fig. 7. Ni et al. revealed that simvastatin could decrease lncRNA SNHG29 expression and, thereby inhibit activation-induced PD-L1 expression, which enhances anti-cancer immunity [124]. Therefore, the use of cholesterol scavengers may significantly enhance the efficacy of immunotherapy [5]. Besides, numerous drugs affect ncRNA-mediated glucose reprogramming to suppress cancer progression. Wu et al. revealed that kaempferol reduced PKM2 expression and thereby inhibited cancer progression by upregulating miR-339-5p [177]. LncRNA UCA1 is documented to contribute to paclitaxel resistance and promote glycolysis by facilitating the expression of HK2 and LDHA [178]. LncRNA FGD5-AS1 promotes glycolysis and 5-FU resistance of CRC cells by acting as a ceRNA for miR-330-3p and upregulating HK2 in CRC. While erlotinib could effectively re-sensitivity CRC to 5-Fu by targeting lncRNA FGD5-AS1 [179].

Importantly, targeting dysregulated ncRNAs by delivering small molecule drugs to the body provides a novel approach for future cancer therapy. Antisense oligonucleotides (ASO) of lncRNA AGPG delivered to patients with ESCC have been demonstrated to effectively limit glycolysis and cell proliferation to inhibit the expression of PFKFB3 [180]. Song et al. demonstrated that siRNA specific to lncRNA HISLA inhibits glycolysis and chemoresistance in breast cancer by mediating the interaction of HIF-1α with prolyl hydroxylase 2 (PHD2) [181]. The study by Yun et al. has identified that applicate inhibition of LINRIS via in vivo-optimized RNA interference (RNAi) and oxaliplatin could effectively suppress glycolysis and cell proliferation in CRC [46]. The reprogramming of energy metabolism mediated by ncRNAs is a potential target for some drugs [124, 177–179]. As a novel therapeutic approach, the combination of small molecule inhibitors with cancer therapy can significantly suppress the progression of cancer [180, 181]. However, the main challenge of their application currently lies in how to precisely target specific cell types. More notably, despite the rapid development of CAR-T cell therapy in non-solid cancers, its treatment currently faces many challenges [182]. Whether lncRNAs can serve as ideal targets to provide new strategies to improve the efficiency of CAR-T cell immune treatment deserves further investigation.

## Conclusion

Energy metabolic reprogramming is a fundamental feature of cancers. Cancer cells reprogram substances such as glucose, lipid and amino acids to satisfy their needs for rapid proliferation and adaptation to external stress, which is driven by dysregulated ncRNAs. Interestingly, numerous studies have suggested that ncRNAs derived from gut microbial and viruses are involved in metabolic reprogramming which may be an emerging direction for future research. Importantly, sufficient studies have demonstrated that alterations in metabolic pathways mediated by ncRNAs are closely associated with the development of cancer treatment tolerance, therefore, the targeted ncRNAs therapy in combination with conventional cancer therapy may be an effective strategy to overcome low response or tolerance of cancer therapy. However, current studies on the metabolism of ncRNAs are mainly focused on glucose and lipids, whereas molecular mechanisms in amino acid metabolism and more systematic research are still required. Moreover, the issues about how to target ncRNAs in specific cells to improve the efficacy of treatment need to be further researched. In conclusion, this study emphasizes the feasibility of targeting lncRNAs and presents new insights into the molecular signaling pathways associated with metabolism.

## Data Availability

Not applicable.

## Abbreviations

3'-UTR: 3’-Untranslated regions
5-Fu: 5-Fluorouracil
ACAC: Acetyl coenzyme A carboxylase
ACAD9: Acyl coenzyme a dehydrogenase-9
ACC: Acetyl coenzyme A carboxylase
ACLY: ATP-citrate synthase
AMPK: AMP-activated protein kinase
ASAP: ATP synthase-associated peptide
ASCL2: Long-chain-fatty-acid-CoA ligase 2
ASCT2: Amino-acid transporter 2
ASO: Antisense oligonucleotides
ASS1: Argininosuccinate synthase 1
ATP: Adenosine triphosphate
BCL6: B-cell lymphoma 6 protein
cccDNA: Covalently closed loop DNA
ceRNA: Competitive endogenous RNA
CircRNAs: Circular non-coding RNAs
CPT-1: Carnitine palmityltransferase-1
CRC: Colorectal cancer
ENO1: Alpha-enolase
EVs: Vesicles
FABPs: Fatty acid binding proteins
FAO: Fatty acid oxidation
FASN: Fatty acid synthase
FATPs: Fatty acid transport proteins
G6PD: Glucose-6-phosphate dehydrogenase
GC: Gastric cancer
GLS: Glutaminase
GLUT1: Glucose transporter1
GPT2: Glutamic–pyruvic transaminase
GSH: Glutathione
GSK3β: Glycogen synthase kinase 3β
HBV: Hepatitis B virus
HCC: Hepatocellular carcinoma
HIF-1α: Hypoxia-inducible factor-1α
HK2: Hexokinase2
HMGCR: HMG-CoA reductase
HSF1: Heat shock factor 1
HSP: Heat shock protein
IDO1: Indoleamine 2, 3-dioxygenase 1
IFN-γ: Interferon-γ
LDHA: Lactate dehydrogenase A
LDLRs: Low-density lipoproteinreceptor superfamily
LncRNAs: Long non-coding RNAs
M1: Classical activated macrophages
M2: Alternatively activated macrophages
MDSCs: Myeloid-derived suppressor cells
miRNA: MicroRNA
mRNA: Messenger RNA
MSCs: Mesenchymal stem cells
mtDNA: Mitochondrial DNA
mTORC: Mammalian target of rapamycin
NAFLD: Non-alcoholic fatty liver disease
NcRNAs: Non-coding RNAs
NK: Natural killer
NPC: Nasopharyngeal carcinoma
NRF: Nuclear respiratory factor
OAZ2: Ornithine decarboxylase antizyme 2
ODC1: Ornithine decarboxylase 1
OXPHOS: Oxidative phosphorylation
PC: Prostate cancer
PDC: Pyruvate dehydrogenase complex
PDK: Pyruvate dehydrogenase kinase
PD-L1: Programmed death ligand-1
PFK-1: Phosphofructokinase-1
PFKFB3: 6-Phosphofructo-2-kinase/fructose-2,6-bisphosphatase 3
PGC-1α: PPARγ coactivator-1α
PGK1: Phosphoglycerate kinase 1
PHD2: Prolyl hydroxylase 2
PHGDH: Phosphoglycerate dehydrogenase
piRNA: PIWI-interacting RNA
PKM2: Pyruvate kinase M2
PPAR: Peroxisome proliferators-activated receptors
PPP: Pentose phosphate pathway
PTEN: Phosphatase and tensin homolog
PTMs: Post-transcriptional modifications
RAI: Radioiodine
RNAi: RNA interference
ROS: Reactive oxygen species
rRNA: Ribosomal RNA
SCD: Stearoyl-CoA desaturase
SDH: Succinate dehydrogenase
snoRNA: Small nucleolar RNA
snRNA: Small nuclear RNA
SQLE: Squalene monooxygenase
SREBP: Sterol-regulatory element binding proteins
SRSF3: Serine and arginine-rich splicing factor 3
STAT3: Signal transducer and activator of transcription 3
TAC: Tricarboxylic acid cycle
TBK1: TANK-binding kinase 1
TDO: Tryptophan 2,3-dioxygenase
TFAM: Mitochondrial transcription factor A
TME: Tumor microenvironment
TNBC: Triple-negative breast cancer
tRNA: Transfer RNA
VLDLR: Very low-density-lipoprotein receptor
YAP: Yes-Associated Protein
